# *NELL2*-*PAX7* Transcriptional Cascade Suggests Activation Mechanism for *RAD52*-Dependent Alternative Lengthening of Telomeres During Malignant Transformation of Malignant Peripheral Nerve Sheath Tumors: Elongation of Telomeres and Poor Survival

**DOI:** 10.3390/biomedicines13020281

**Published:** 2025-01-23

**Authors:** Jungwoo Lee, Eunji Choi, Hyoju Kim, Young-Joon Kim, Seung Hyun Kim

**Affiliations:** 1 Interdisciplinary Program of Integrated OMICS for Biomedical Science, The Graduate School, Yonsei University, Seoul 03722, Republic of Korea; ljw90607@hotmail.com (J.L.); yatookk@gmail.com (E.C.); khyojoo01@gmail.com (H.K.); yjkim@yonsei.ac.kr (Y.-J.K.); 2 Department of Orthopaedic Surgery, Yonsei University College of Medicine, 50-1, Yonsei-Ro, Seodaemun-gu, Seoul 03722, Republic of Korea

**Keywords:** telomere, *NELL2*, *PAX7*, RAD52-dependent ALT, MPNST

## Abstract

**Background**: In eukaryotes with a double-stranded linear DNA genome, the loss of terminal DNA during replication is inevitable due to an end-replication problem; here, telomeres serve as a buffer against DNA loss. Thus, the activation of the telomere maintenance mechanism (TMM) is a prerequisite for malignant transformation. **Methods**: We compared neurofibroma (NF, benign) and malignant peripheral nerve sheath tumors (MPNSTs) occurring in the same patient with type 1 neurofibromatosis, where each NF–MPNST pair shared the same genetic background and differentiation lineage; this minimizes the genetic bias and contrasts only those changes that are related to malignant transformation. A total of 20 NF–MPNST pairs from 20 NF1 patients were analyzed. Whole-transcriptome sequencing (WTS) was conducted to profile the transcriptional relationship, and whole-genome sequencing (WGS) was performed to measure the telomere length. **Results**: We identified 22 differentially expressed genes (DEGs) during the malignant transformation of MPNSTs. Among them, *NELL2* activated *PAX7*, which sequentially activated *RAD52*, the recombinase of RAD52-dependent alternative lengthening of telomeres (ALT). *RAD52* elongated MPNSTs–telomeres (*p* = 0.017). Otherwise, neither *NELL2* nor *PAX7* affected telomere length (*p* = 0.647 and *p* = 0.354, respectively). *RAD52* increased MPNSTs–telomeres length, independently of *NELL2* and *PAX7* in multiple analyses (*p* = 0.021). The group with increased telomere length during the malignant transformation showed inferior overall survival (OS) (HR = 3.809, *p* = 0.038) to the group without increased telomere length. Accordingly, the group with increased *PAX7* showed inferior OS (HR = 4.896, *p* = 0.046) and metastasis-free survival (MFS) (HR = 9.129, *p* = 0.007) in comparison to the group without increased *PAX7*; the group with increased *RAD52* showed inferior MFS (HR = 8.669, *p* = 0.011) in comparison to the group without increased *RAD52*. **Conclusions**: We suggest that the *NELL2*-*PAX7* transcriptional cascade activates RAD52-dependent ALT to increase telomere length during the malignant transformation of MPNSTs, resulting in a poor prognosis.

## 1. Introduction

Telomeres are essential structures on the ends of the chromosomes of eukaryotes with double-stranded linear DNA genomes, and they assist in overcoming end-replication problems [1]. Due to the 5′ to 3′ unidirectionality of DNA polymerization, an Okazaki fragment with an RNA primer is required on lagging strands [2] and DNA loss corresponding to the terminal RNA primer on the lagging strand is inevitable [1]. The cumulative effects of DNA loss during DNA replication are responsible for replicative senescence. Telomeres are located at both ends of the chromosomes and serve as a buffer against DNA loss through DNA replication and they maintain genomic stability.

The Fédération Nationale des Centres de Lutte Contre Le Cancer (FNCLCC) grading system consists of three categories: tumor differentiation, mitotic count, and tumor necrosis [3,4]. The mitotic count is the underlying determinant for tumor dedifferentiation and necrosis, and unlimited mitosis is the most fundamental cellular feature of malignant tumors. The activation of the telomere maintenance mechanism (TMM) is essential for overcoming the Hayflick limit and achieving immortalization. The first identified TMM was a type of reverse transcriptase, later called telomerase [5,6]. Human telomerase is composed of TERT, TERC, DKC1, and TEP1. Shelterin, which includes TRF1, TRF2, TPP1, POT1, TIN2, and RAP1, supports telomerase recruitment to telomeres and the protection of telomeres by forming secondary telomeric structures, such as T- and D-loops [7]. The H/ACA snoRNP complexes associated with DKC1, NHP2, NIP10, GAR1, and NAF1, together with hTERT, constitute the catalytic core of telomerase [8]. Approximately 85 to 90% of human cancers exhibit increased telomerase activity, whereas the remaining 10 to 15% lack telomerase activity and adopt the alternative lengthening of telomeres (ALT) [9,10,11]. In addition to the ALT, which is based on homologous recombination (HR), break-induced replication (BIR) [12,13,14] and mitotic DNA synthesis (MiDAS) [13] have also been reported to be associated mechanisms by which RAD52-dependent ALT overcomes deficient telomerase activity.

Type 1 neurofibromatosis (NF1) is an autosomal dominant disorder including a tumor suppressor gene, *neurofibromin 1*, located on 17q11.2, which encodes a GTPase-activating protein involved in the RAS/MAPK pathway [15]. The average global prevalence of NF1 is approximately 1 in 3000 individuals, and individuals harboring NF1 have a life expectancy of 8 to 21 years less than the general population [16]. The most fatal clinical manifestation of NF1 is a malignant peripheral nerve sheath tumor (MPNST), which involves the malignant transformation of preexisting neurofibromas. The cumulative lifetime risk for a malignant transformation in NF1 patients is estimated to be approximately 8–13%. MPNSTs are rare sarcomas that account for approximately 5–10% of all soft-tissue sarcomas. Notably, both telomerase activity and ALT have been observed in MPNSTs, instead of the ALT replacing telomerase activity [17,18], which indicates that MPNSTs are optimal tumors in comprehensive analyses of the TMM.

The ALT is a major TMM of malignant tumors originating from the mesenchyme and neuroepithelium. It has been reported that there are two major independent ALT pathways with distinct mechanisms and times of action: the RAD51- and RAD52-dependent ALT pathways. RAD51-dependent ALT promotes semiconservative homologous recombination (HR) with high fidelity [19], whereas RAD52-dependent ALT promotes conservative HR as well as breaks-induced replication-related DNA synthesis [20,21]. This conservative DNA synthesis mechanism is observed only in the late phase of the cell cycle, G2/M [12,13]. These findings suggest that RAD51- and RAD52-dependent ALT are regulated independently of each other. Since the molecular mechanisms of the ALT share a DNA repair process and DNA damage signals, DNA damage signaling has been characterized as an initiator of the ALT. Indeed, the association between the DNA damage response and RAD51-dependent ALT has been well documented [22,23]. However, the activation signals for RAD52-dependent ALT have not been elucidated and remain an outstanding question in telomere biology.

**Figure 1:**
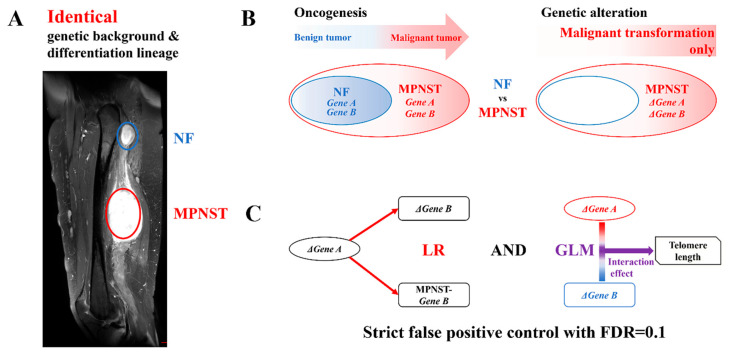
Schematic illustrations of this study. (**A**) An example of a T2 sagittal MR image of a thigh showing an NF (blue circle) and an MPNST (red circle) in one patient with NF1. (**B**) Since the MPNSTs associated with NF1 arise from preexisting NFs, each NF–MPNST pair shares the same genetic background and differentiation lineage. In each NF–MPNST pair comparison, only the malignant transformation-related changes are determined by structurally subtracting the changes from the normal cell to the benign tumor. (**C**) A combined statistical analysis method is utilized, where both the interaction effects on the telomere length and the transcriptional relationships for all the gene pairs are analyzed to distinguish only the TMM-related transcriptional relationships and filter out those that are unrelated. To minimize false positives in the statistical results, extremely high *p*-value controls (FDR = 0.1) are applied to all the statistical results. MR, magnetic resonance; NF, neurofibroma; MPNST, malignant peripheral nerve sheath tumor; NF1, type 1 neurofibromatosis; FDR, false discovery rate.

New strategies were employed in this study as follows. First, we designed a genetic analysis model conceptually similar to a “subtractive cDNA library”. We focused on the MPNSTs associated with NF1 and compared the MPNSTs and NFs from the same NF1 patient (Figure 1A). Since the MPNSTs associated with NF1 arose from preexisting NFs, each NF–MPNST pair in this model shared the same genetic background and differentiation lineage, minimizing the bias due to genetic variation and differentiation. Furthermore, the comparison of each NF–MPNST pair contrasted only the malignant transformation-related changes, structurally subtracting those changes from the normal cell to the benign tumor (Figure 1B). Second, we designed a combined statistical analysis method in which we analyzed the interaction effects on the telomere length as well as the transcriptional relationships for all the gene pairs, distinguishing the TMM-related transcriptional relationships and filtering out those that were unrelated under a strict false-positive control with FDR = 0.1 (Figure 1C). A total of 20 NF–MPNST pairs from 20 NF1 patients were analyzed to evaluate the activation of the TMM during an MPNST malignant transformation. Whole-transcriptome sequencing (WTS) was performed to profile the genome-wide gene expression and whole-genome sequencing (WGS) was performed to measure the telomere length.

The dysregulation of the major transcription factors (MTFs) involved in development may mediate the aberrant gene expression associated with oncogenesis in cancer cells [24]. Although they are essential for differentiation during development (orthodox function), the unexpected activation of MTFs in cancer cells has recently been reported to induce the expression of oncogenes in various cancers (unorthodox function) (Figure 3A). We focused on the paired box (PAX) genes because they are essential for neural development and we hypothesized that differentially expressed genes (DEGs) activate PAX genes and turn on their unorthodox function, driving oncogenesis during the malignant transformation of MPNSTs. As a result, we suggest that the *NELL2*-*PAX7* transcriptional cascade increases telomere length during the malignant transformation of MPNSTs by activating RAD52-dependent ALT, which results in poor survival.

## 2. Materials and Methods

### 2.1. Patients and Samples

We analyzed 20 patients with NF1 who underwent surgery for both NFs and MPNSTs from November 2000 to July 2017. The clinical characteristics of the patients and tumors (NFs and MPNSTs) are described in Supplementary Material S1. Only 4 of the NF–MPNST pairs were derived from fresh-frozen tissues, and the remaining 16 NF–MPNST pairs were derived from formalin-fixed paraffin-embedded (FFPE) tissues. The FFPE samples were prepared as slides for histopathological qualification.

### 2.2. Whole-Genome Sequencing (WGS)

The integrity of the genomic DNA was checked by agarose gel electrophoresis and gDNA was quantified using Quant-IT PicoGreen (Invitrogen, Waltham, MA, USA). The sequencing libraries were prepared according to the instructions for the TruSeq DNA Nano Library Prep Kit. Briefly, genomic DNA (100 ng) was fragmented using adaptive-focused acoustic technology (Covaris) and end-repaired to create 5-phosphorylated blunt-ended dsDNA molecules. Following end-repair, the size of the DNA was selected via a bead-based method. DNA fragments were further processed by the addition of a single ‘A’ base, and ligation with TruSeq indexing adapters. The purified libraries were quantified using qPCR according to the qPCR Quantification Protocol Guide (KAPA Library Quantification Kit for Illumina sequencing platforms) and quantified using an Agilent Technologies 2200 TapeStation (Agilent Technologies, Santa Clara, CA USA). Then, paired-end (2 × 150 bp) sequencing was performed by Macrogen (Seoul, Korea) using the NovaSeq platform (Illumina). WGS was used to measure the telomere length of 38 samples (19 each for NFs and MPNSTs).

### 2.3. Whole-Transcriptome Sequencing (WTS)

The total RNA concentration was calculated by Quant-IT RiboGreen (Invitrogen, Waltham, MA, USA). To determine the DV200 (% of RNA fragments > 200 bp) value, samples were run on a TapeStation RNA ScreenTape platform (Agilent). Total RNA (100 ng) was subjected to sequencing library construction using a TruSeq RNA Access Library Prep Kit (Illumina, San Diego, CA, USA) according to the manufacturer’s protocols. Briefly, total RNA was first fragmented into small pieces using divalent cations at an elevated temperature. The cleaved RNA fragments were copied into first-strand cDNA using SuperScript II reverse transcriptase (Invitrogen, #18064014) and random primers, followed by second-strand cDNA synthesis using DNA Polymerase I, RNase H, and dUTP. These cDNA fragments then underwent an end-repair process, involving the addition of a single ‘A’ base, and the ligation of adapters. These products were then purified and enriched via PCR to create a cDNA library. All libraries were normalized, and six libraries were pooled into a single hybridization/capture reaction. The pooled libraries were incubated with a cocktail of biotinylated oligos corresponding to coding regions of the genome. The targeted library molecules were captured via hybridized biotinylated oligo probes using streptavidin-conjugated beads. After two rounds of hybridization/capture reactions, the enriched library molecules were subjected to a second round of PCR amplification. The captured libraries were quantified using a KAPA Library Quantification Kit for the Illumina sequencing platforms according to the qPCR Quantification Protocol Guide (KAPA BIOSYSTEMS, Wilmington, MA, USA, #KK4854), and qualified using the TapeStation D1000 ScreenTape platform (Agilent Technologies, # 5067–5582). The indexed libraries were then submitted to sequencing on the Illumina NovaSeq platform (Illumina) and paired-end (2 × 100 bp) sequencing was performed by Macrogen. WTS was used to profile the transcriptomes of 40 samples (20 each for NFs and MPNSTs). All TPM values (NFs and MPNSTs) of the 131 genes evaluated in this study are shown in Supplementary Material S2.

### 2.4. DEG Selection

The raw RNA-seq data (fastq) of all the samples were mapped using STAR (v2.7.3) and quantified by individual genes with a normalized count value, transcripts per million (TPM), using RSEM (v.1.2.31). Noncoding genes, including pseudogenes and long noncoding RNA, were excluded from the analysis and only coding genes were used (20,315 coding genes). The expression value was log2 transformed (log_2_(TPM + 1)), where the entire expression profile was proportionally transformed, and the 0 conversions were corrected by adding 1 to the substitution. By comparing 20 NF–MPNSTs, the DEGs between the NFs and MPNSTs were selected on the basis of an average fold-change greater than two and a *t*-test *p*-value < 0.001. To achieve a fairly high confidence level in DEG selection, we adopted a *p*-value < 0.001 to select the most reliable and appropriate number of genes. Using the above filtering criteria, 22 significant DEGs were selected. Statistical testing for each gene was achieved using paired *t*-tests and a *p*-value < 0.05 was considered statistically significant.

### 2.5. Measuring the Length of Telomeres

Telomeric reads were calculated using TelomereHunter [25]. The program extracts telomeric reads with nonconsecutive repeats, and the extracted telomeric reads are categorized according to their alignment coordinates and mapping quality. The reads are classified into different telomeric regions. The telomere content is given as the fraction of intratelomeric reads per million reads. The program also accounts for GC bias and GC is used to correct the telomeric content by dividing the intratelomeric reads by the GC number of reads, with a GC content between 48% and 52%. In this study, GC-corrected telomeric reads were used.

### 2.6. Statistical Analysis

Statistical analyses were performed using SPSS (SPSS, Inc., Chicago, IL, USA). Transcriptional relationships were analyzed with LR. For each gene pair, transcriptional activation of the B gene by the A gene was defined as follows: the ΔA transcripts during NF–MPNST transformation were correlated with both the ΔB transcripts and the MPNST-B transcripts, where the significance of the LR results between the ΔA transcripts and the ΔB transcripts was confirmed using the Benjamini–Hochberg procedure. The interaction effects of genes A and B on the telomere length were also analyzed with a GLM (Figure 1C). Correlations with the histologic grade were evaluated using logistic regression, and MFS and OS were evaluated using Cox regression.

## 3. Results

### 3.1. Differentially Expressed Genes During MPNST Malignant Transformation

In this study, 20 NF–MPNST pairs from 20 NF1 patients were analyzed. The patient demographics and tumor characteristics are described in Table 1. The mean latency to MPNST presentation was 36.55 years, and 30.0% of the patients were in the metastatic stage at MPNST presentation. The OS rate was 35.0%, and the metastasis rate was 71.4%. In terms of the tumors, 50% of the MPNSTs were high-grade, with an FNCLCC grade of 3. There were no significant differences in tumor sizes (*p* = 0.430). There was a significant difference in the telomere length between the NFs (999.96 ± 423.69 intratel_reads × 1,000,000/total_reads_with_tel_gc) and MPNSTs (727.32 ± 490.75 intratel_reads × 1,000,000/total_reads_with_tel_gc) in the paired *t*-tests (*p* = 0.043) (Table 1). Although there were statistical differences between the NFs and MPNSTs telomere lengths, the differences did not show a clear distinction, suggesting that MPNTs achieve immortalization through adequate TMM activation.

**Figure 2:**
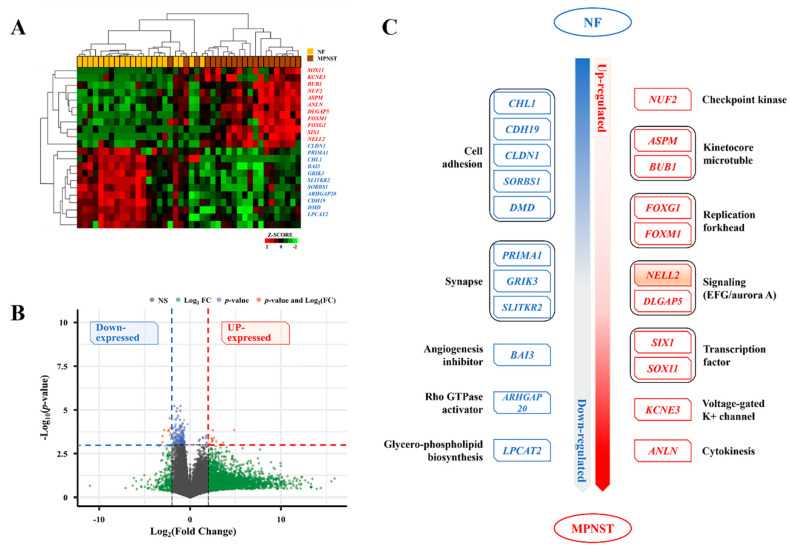
Twenty-two DEGs during malignant transformation of MPNSTs. (**A**) Z-score heatmap and (**B**) volcano plot for 22 DEGs during malignant transformation of MPNSTs. Eleven upregulated DEGs are shown in red, whereas the eleven downregulated DEGs are shown in blue. (**C**) Classification diagram of 22 DEGs according to their biological function. DEG, differentially expressed gene.

Twenty-two differentially expressed genes (DEGs) were identified from the WTS during the MPNST malignant transformation by selecting the genes with an average fold-change of greater than twofold. The Z-score heatmap (Figure 2A) and volcano plot (Figure 2B) are illustrated. All the NF and MPNST groups do not have the same profile, and the level of gene expression appears to be different in the heatmap. This is caused by the fact that the cellular heterogeneity of tissues may vary slightly from sample to sample, which may lead to slight differences in the gene expression profiles. Eleven upregulated genes were mainly classified as related to (1) cell signaling—*NELL2* and *DLGAP5*; (2) cytokinesis—*ASPM* and *BUB1*; (3) forkhead-box transcription factors—*FOXG1* and *FOXM1*; and (4) transcription factors:—*SIX1* and *SOX11*, whereas eleven downregulated genes were mainly classified as related to (1) cell adhesion—*CHL1*, *CDH19*, *CLDN1*, *SORBS1*, and *DMD*; and (2) synapse-related—*PRIMA1*, *GRIK3*, and *SLITKR2*. The details of the 22 DEGs are schematically shown in Figure 2C.

Among the upregulated DEGs, BUB1 promotes telomere replication during the S phase in HeLa cells [26]. Although the NELL2-Robo3 complex is involved in the activation of axonal guidance [27], NELL2 is also involved in various cancers, for instance, the NELL2/cdc42-BAF complex in Ewing’s sarcoma cells [28], fat mass and obesity-associated protein (FTO)/E2F1/NELL2 in non-small-cell lung cancer (NSCLC) [29], and NELL2/N-cadherin in embryonic carcinoma cells [30]. FOXM1 is highly expressed and a marker of poor prognosis in several cancers [31], including MPNST [32]. SOX11 is also highly expressed in nervous system neoplasms [33]. Among the downregulated DEGs, PRIMA1 has been reported to be a tumor suppressor by restoring mutant p53 [34] and inducing tumor cell death [35]. DMD is also a tumor suppressor in sarcomas, hematologic malignancies, and nervous system tumors [36]. SORB1 suppression promotes lung adenocarcinoma [37].

### 3.2. NELL2 Activates PAX7 During Oncogenesis of MPNST

**Figure 3:**
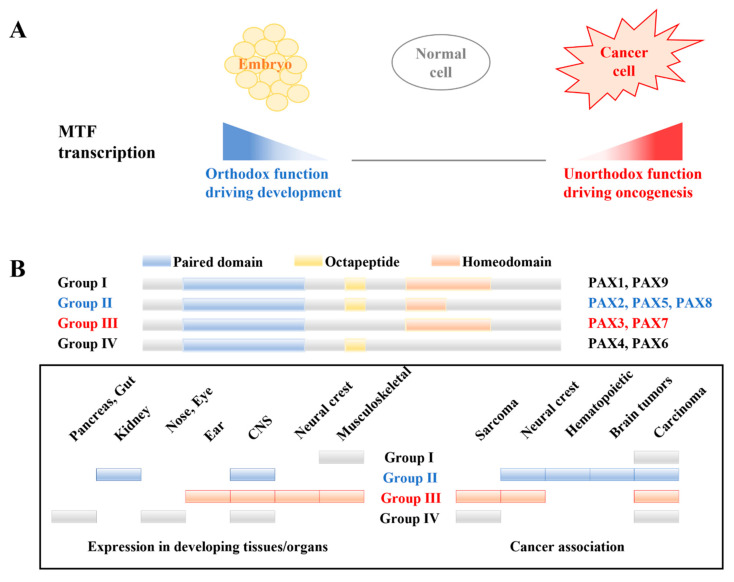
PAX genes as MTFs with unorthodox function. (**A**) Unexpected transcription of MTFs in cancer cells leads to the expression of oncogenic proteins, rather than normal developmental proteins. (**B**) PAX genes are classified into four groups according to the paired domain, octapeptide, and homeodomain. Each group plays important roles in the development and oncogenesis of various tissues, especially neural tissues. Group III: PAX3 and PAX7 have functions in neuromuscular tissue development and the oncogenesis of sarcoma. MTF, major transcription factor.

The paired box (PAX) genes encode transcription factors with highly conserved N-terminal DNA-binding domains, known as paired domains, which have been reported to be important in neural development and oncogenesis. In addition to their orthodox functions in normal neural development, several studies have described their unorthodox oncogenic functions in various cancers (Figure 3A). Mutations in the PAX genes are associated with congenital human diseases related to eye development and deafness, including Aniridia and Peter’s anomaly (*PAX6*) and Waardenburg syndrome (*PAX3*), suggesting that the PAX genes play a central role in the development of the nervous system [38]. Moreover, the PAX genes have also been reported to be involved in oncogenesis [39]. The nine PAX genes are subclassified into four groups according to their homeodomain and octapeptide domain: Group I (PAX1 and PAX9), Group II (PAX2, PAX5, and PAX8), Group III (PAX3 and PAX7), and Group IV (PAX4 and PAX6). Group III contributes to sarcomas (Ewing’s sarcoma and rhabdomyosarcoma) and neural crest-derived tumors [40,41] (Figure 3B).

We analyzed both the transcriptional relationship and the interaction effects on the telomere length for 198 DEG-PAX gene pairs, comprising 22 DEGs and 9 PAX genes. The transcriptional activation of a PAX gene by a DEG was defined as the significant positive correlation of the ΔDEG transcript with both the Δ*PAX* transcript and the MPNST*-PAX* transcript according to a linear regression (LR). In addition, to screen for only TMM-related transcriptional activation, the interaction effects of each gene pair on the telomere length (MPNST–telomere) were also analyzed using a generalized linear model (GLM). All 594 *p*-values from the LRs for Δ*PAX* and MPNST-*PAX* and the GLM were verified to control the false discovery rate (FDR) using the Benjamini-Hochberg (B-H) procedure, with a false discovery rate (FDR) of 0.1. Conclusively, the transcriptional activation of the TMM activation pathway was defined as a significant result for both the LR and GLM, following verification by the B-H procedure (Figure 1C). *NELL2* transcriptionally activates *PAX7* and *SORBS1* transcriptionally activates *PAX5* (Supplementary Material S3). The transcriptional activation of PAX7 by the 22 DEGs is summarized in Table 2.

### 3.3. PAX7 Activates RAD52-Dependent ALT During Oncogenesis of MPNST

An in-depth search of the literature revealed 100 genes that play important roles in the TMM. These genes were classified into four main categories based on their function in the TMM: (1) telomere machinery, (2) telomere topology, (3) regulatory genes involved in the DNA damage response and cell cycle/checkpoint, and (4) TMM-effector genes. The abbreviations and details for the 100 genes are listed in Supplementary Material S1. To evaluate the downstream regions of *PAX7* and *PAX5*, we analyzed both the transcriptional relationship and interaction effects on the telomere length of 200 pairs comprising these 2 genes and 100 TMM-related genes. In addition, we evaluated the process of TMM activation caused by DNA damage signals. The DNA damage markers *H2AFX*, *PARP1*, *P53*, and *RB1* were also analyzed in the same way as above for 396 pairs comprising these 4 genes and the remaining 96 TMM-related genes. Finally, all 1788 *p*-values from the two LRs and GLM for the 596 pairs were verified to control the false discovery rate (FDR) by the Benjamini-Hochberg (B-H) procedure (FDR = 0.1). *PAX7* activates *RAD52* and *SLX4IP* [24], whereas *PAX5* has no effect on TMM activation. In the DNA damage signaling pathway, *H2AFX* activates *RAD51* and *P53* activates *FEN1*, but those results did not pass verification by the B-H procedure. (Supplementary Material S4).

### 3.4. RAD52-Dependent ALT Elongates Telomeres During Oncogenesis of MPNST, Which Results in Poor Survival

We evaluated the biological and clinical manifestations of RAD52-dependent ALT induced by the *NELL2*-*PAX7* transcriptional cascade. We first evaluated their effects on telomere length during the malignant transformation. The correlations between Δ*NELL2*, Δ*PAX7*, Δ*RAD52*, Δ*H2AFX*, and Δ*RAD51* and the telomere length are below. The Δtelomere length is the telomere length change during a malignant transformation from a benign (NF) to malignant tumor (MPNST) and the MPNST–telomere length represents the telomere length in an MPNST. They were evaluated using an LR analysis. *RAD52* was the only gene that correlated with the MPNST–telomere length in the simple analysis (B = 114.894, *p* = 0.017), and *RAD52* was independent of *NELL2* and *PAX7* in the multiple analysis (B = 161.989, 0.021). Although RAD51-dependent ALT is also a major component of TMM, RAD51 was not associated with the telomere length (B = −23.229, *p* = 0.633 for Δtelomere length and B = −34.669, *p* = 0.603 for MPNST–telomere length). This finding strongly suggests that *NELL2*-*PAX7*-induced RAD52-dependent ALT plays a dominant role in telomere maintenance and overcomes replicative senescence to achieve the malignant transformation of MPNST (Table 3).

**Figure 4:**
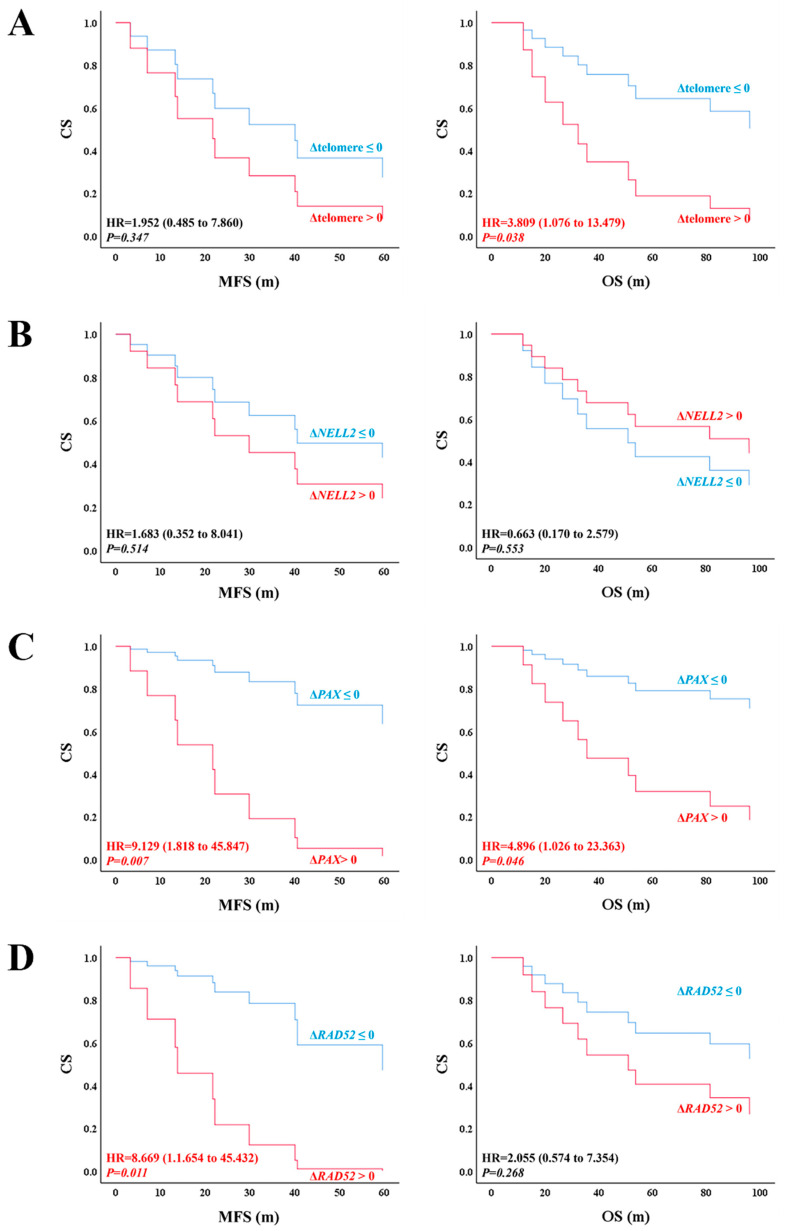
Survival analysis of RAD52-dependent ALT induced by *NELL2*-*PAX7* transcriptional cascade. MFS and OS are evaluated using Cox regression between the group with increased transcripts (Δfactor > 0) and the group with transcripts that were not increased (Δfactor ≤ 0) during the malignant transformation. (**A**) Telomere length, (**B**) *NELL2*, (**C**) *PAX7*, and (**D**) *RAD52* are analyzed. The red lines represent the increased group (Δfactor > 0) and the blue lines represent the not increased group (Δfactor ≤ 0). MFS, metastasis-free survival; OS, overall survival; CS, cumulative survival; m, months; HR: hazard ration.

To evaluate the association between telomere length and prognosis, we divided all the factors into two groups according to their changes during the malignant transformation: increased (Δtranscript > 0) and not increased (Δtranscript ≤ 0) groups. The overall survival (OS) and metastasis-free survival (MFS) were compared between the two groups using a Cox regression. The group with increased telomere length showed an inferior OS to the group without increased telomere length (Figure 4A right panel, HR = 3.809 (1.076 to 13.479), *p* = 0.038), suggesting that achieving telomere maintenance leads to a poor prognosis. Additionally, the group with an increased *PAX7* transcript showed an inferior MFS (Figure 4C left panel, HR = 9129, *p* = 0.007) and OS (Figure 4C right panel, HR = 4.896, *p* = 0.046) in comparison with the group without an increase in the *PAX7* transcript. The group with an increased *RAD52* transcript showed an inferior MFS to the group without an increased *RAD52* transcript (Figure 4A left panel, HR = 8.669 (1.654 to 45.432), *p* = 0.011). However, *NELL2* was not associated with survival (Figure 4).

## 4. Discussion

We conducted this study with smart strategies. First, we designed a new analysis model optimized for the study of malignant transformation: we compared NFs and MPNSTs in a single NF1 patient. Since MPNSTs develop from preexisting NFs, they must have the same genetic background, which minimizes the genetic bias and the comparisons of the NF–MPNST pairs indicate only the changes that develop during the malignant transformation. Second, we designed a combined statistical analysis method: we analyzed the interaction effects on the telomere length and the transcriptional relationship to distinguish only the TMM-related transcriptional relationships and filter out those that are unrelated. Using these strategies, we suggested that the *NELL2*-*PAX7* cascade activates RAD52-dependent ALT during the malignant transformation of MPNSTs. This answers an outstanding question in telomere biology: what signal activates RAD52-dependent ALT? In addition, we also determined the biological and clinical manifestations of RAD52-dependent ALT. RAD52-dependent ALT activated by the *NELL2*-*PAX7* transcriptional cascade increases telomere length, leading to a poor prognosis for the MPNSTs.

Transcriptional activation is the most universal and fundamental process in gene activation and is involved in numerous signaling pathways and developmental processes. However, although studies of the TERT promoter [42] and transcriptional regulation of the TMM have been attempted [43,44], the transcriptional activation of the TMM is still unknown. No notable TMM activation signals have been identified, except for those related to DNA damage. Under these challenging conditions, we have suggested a new *NELL2*-*PAX7* transcriptional cascade that activates RAD52-dependent ALT. This discovery could provide a new systematic understanding of the overall architecture of the TMM activation process, providing insights into the fundamental pathways for TMM activation via the transcriptional cascades. Although *PAX7* is known to be essential for neural development (orthodox functions), it has been reported to play unorthodox functions in oncogenesis. At the molecular level, there are similarities between its orthodox and unorthodox functions. A major function of *PAX7* during normal development is to maintain cell proliferation with self-renewal, which is comparable to overcoming the replicative senescence during a malignant transformation. Additionally, resistance to apoptosis and cell migration are required for both normal development and oncogenesis (metastasis). Therefore, the unorthodox functions of *PAX7* may be considered a reinvocation of their orthodox functions during oncogenesis, albeit with different biological consequences.

### Limitations of This Study

Despite these results, there are some limitations that should be considered. First, functional validation through a molecular biology experiment was not performed. Since this study was based on the changes in mRNA transcripts rather than protein–protein interactions, we were unable to analyze the genes for epigenetic changes, such as phosphorylation and SUMOylation (small ubiquitin-related modifier). In this study, *H2AFX* showed no association with *ATM* or *ATR*, which encode kinases and convert H2AX to its active form, γH2AX, which is a DNA damage marker. *ATRX*/*DAXX*, which belongs to the SWI/SNF family, suppresses the ALT by depositing H3F3A onto telomeres and resolving G-quadruplex (G4) loops [45]. *ATRX* encodes an ATP-dependent helicase that undergoes cell cycle-dependent phosphorylation and *DAXX* encodes a potent transcriptional repressor that binds to SUMOylated transcription factors. These genes showed no association with the *NELL2*-*PAX7* cascade in this study. In addition, the *SMC5*/*SMC6* complex and *NSMCE2*, which are representative examples of SUMOylation regulation in the TMM [46], also showed no significant interactions in this study. Therefore, in this study, the significance of DNA damage signaling (MDC1-ATM/ATR-CHEK1 pathway), the telomere topology (ATRX/DAXX), and telomere recruitment to the ALT-associated promyelocytic leukemia nuclear body (APB) (SMC complex and NSMCE2) may have been partly obscured and underestimated due to methodological reasons. The findings in this study need to be further validated at the protein–protein interaction level by wet-laboratory research.

Second, this study was conducted entirely through statistical analyses and consisted of a relatively small number of cases, which may be a clear weakness. However, NF1-associated MPNST is very rare, with an incidence of approximately 0.0005% in the general population. Therefore, it is difficult to secure a large cohort. To maximize the reliability of the statistical results of this study, we focused on false-positive control. To minimize false positives in the statistical results, extremely high *p*-value controls (FDR = 0.1) were applied to all the statistical results in this manuscript.

## Figures and Tables

**Table 1:** Patient demographics and tumor characteristics.

		NF	MPNST	*p*
Patient demographics				
Age at diagnosis of MPNST (years, mean ± SD) (Min–Max)		36.55 ± 14.58 (16–71)		-
Sex n (%)	Male	10 (50.0)		-
	Female	10 (50.0)		-
AJCC stage ^1^ n (%)	I	-	4 (20.0)	-
	II and IIIA		10 (50.0)	
	IIIB and IV (Metastatic)		6 (30.0)	
5-Year Survival n (%)	OS ^2^	-	7 (35.0)	-
	DOD		10 (50.0)	
	DOC		3 (15.0)	
Metastasis n (%)	Free	-	4 (28.6)	
	Positive		10 (71.4)	
Tumor characteristics				
Size (cm, mean ± SD) ^2^		5.31 ± 5.57	7.44 ± 3.49	0.430
MPNST histologic grade (FNCLCC) n (%)	1	-	4 (20.0)	-
	2		6 (30.0)	
	3		10 (50.0)	
Location ^3^ n (%)	Visceral	0 (0)	2 (10.0)	0.625
	Axial	5 (25.0)	5 (25.0)	
	Extremity	15 (75.0)	13 (65.0)	
Telomere length ^4^ (mean ± SD)		999.96 ± 423.69	727.32 ± 490.75	0.043

^1^ 8th edition; ^2^ paired t-test; ^3^ Fisher’s exact test; ^4^ paired t-test, measured by TelomereHunter, tel_content = intratel_reads × 1,000,000/total_reads_with_tel_gc; Abbreviations: NF, neurofibroma; MPNST, malignant peripheral nerve sheath tumor; SD, standard deviation; Min, minimum; Max, maximum; AJCC, American Joint Committee on Cancer; OS, overall survival; DOD, died of disease; DOC, died of other cause; FNCLCC, Fédération Nationale des Centres de Lutte Contre Le Cancer.

**Table 2:** *NELL2* transcriptionally activates *PAX7* during oncogenesis of MPNST.

		*PAX7*					
		Linear Regression				Generalized Linear Model (MPNST–Telomere)	
		Δ*PAX7*		MPNST-*PAX7*			
		B (95% CI)	*p*	B (95% CI)	*p*	B (95% CI)	*p*
Upregulated DEGs	*NUF2*	0.485 (0.158 to 0.813)	0.006	0.487 (0.159 to 0.815)	0.006	116.359 (5.496 to 227.221)	0.040
	*ASPM*	0.493 (0.166 to 0.821)	0.005	0.493 (0.165 to 0.822)	0.005	0.996 (−112.384 to 114.376)	0.986
	*SOX11*	0.346 (−0.506 to 1.198)	0.405	0.341 (−0.514 to 1.195)	0.413	−300.907 (−451.046 to −150.769)	0.000
	*BUB1*	0.570 (0.274 to 0.866)	0.001	0.571 (0.274 to 0.867)	0.001	109.500 (−80.466 to 299.466)	0.259
	*ANLM*	0.216 (−0.023 to 0.454)	0.074	0.215 (−0.024 to 0.454)	0.075	−56.385 (−135.771 to 23.002)	0.164
	*KCNE3*	0.052 (−0.545 to 0.649)	0.857	0.051 (−0.547 to 0.650)	0.806	−84.948 (−242.311 to 72.415)	0.290
	*FOXM1*	0.671 (0.300 to 1.041)	0.001	0.672 (0.300 to 1.043)	0.001	−12.323 (−153.227 to 129.581)	0.864
	*NELL2*	0.335 (0.167 to 0.503)	0.001	0.335 (0.167 to 0.504)	0.001	123.100 (42.249 to 203.951)	0.003
	*FOXG1*	0.391 (0.172 to 0.610)	0.001	0.392 (0.172 to 0.611)	0.001	81.632 (−73.011 to 236.275)	0.301
	*DLGAP5*	0.248 (−0.153 to 0.650)	0.211	0.249 (−0.154 to 0.651)	0.210	85.342 (−57.173 to 227.856)	0.241
Downregulated DEGs	*SIX1*	0.223 (−0.055 to 0.500)	0.109	0.221 (−0.057 to 0.500)	0.112	−41.602 (−100.271 to 17.067)	0.165
	*GRIK3*	0.057 (−0.201 to 0.316)	0.647	0.057 (−0.202 to 0.316)	0.648	−115.882 (−212.860 to −18.904)	0.019
	*CHL1*	0.043 (−0.212 to 0.298)	0.727	0.044 (−0.212 to 0.300)	0.722	114.147 (46.262 to 182.032)	0.001
	*CLDN1*	0.147 (−0.514 to 0.087)	0.646	0.142 (−0.520 to 0.804)	0.657	−19.451 (−156.377 to 117.474)	0.781
	*BAI3*	0.116 (−0.244 to 0.475)	0.508	0.116 (−0.244 to 0.476)	0.507	23.655 (−132.529 to 179.840)	0.767
	*SORBS1*	−0.059 (−0.413 to 0.295)	0.729	−0.059 (−0.413 to 0.296)	0.731	−22.310 (−136.696 to 92.076)	0.702
	*ARHGAP20*	0.229 (−0.246 to 0.703)	0.325	0.230 (−0.245 to 0.705)	0.322	40.714 (−54.309 to 135.737)	0.401
	*PRIMA1*	−0.205 (−0.533 to 0.123)	0.206	−0.206 (−0.534 to 0.123)	0.205	−92.957 (−174.910 to −11.005)	0.026
	*LPCAT2*	0.083 (−0.157 to 0.322)	0.478	0.082 (−0.158 to 0.322)	0.480	−36.528 (−129.540 to 56.484)	0.441
	*CDH19*	−0.088 (−0.332 to 0.156)	0.460	−0.087 (−0.331 to 0.157)	0.464	24.981 (−82.583 to 132.545)	0.649
	*DMD*	−0.093 (−0.424 to 0.238)	0.562	−0.093 (−0.425 to 0.239)	0.564	−47.753 (−191.314 to 95.808)	0.514
	*SLITRK2*	0.178 (−0.321 to 0.676)	0.464	0.178 (−0.321 to 0.678)	0.463	10.067 (−129.352 to 149.486)	0.887

Abbreviations: CI, confidence interval.

**Table 3:** RAD52-dependent ALT elongates telomere length during oncogenesis of MPNST.

		ΔTelomere				MPNST–Telomere			
		Simple		Multiple		Simple		Multiple	
		B (95% CI)	*p*	B (95% CI)	*p*	B (95% CI)	*p*	B (95% CI)	*p*
*NELL2*-*PAX7* cascade	*NELL2*			11.182 (−113.352 to 135.716)	0.851			−21.980 (−125.042 to 81.083)	0.656
	*PAX7*			−199.533 (−517.046 to 117.981)	0.200			−75.310 (−338.079 to 187.459)	0.550
	*RAD52*			157.857 (−3.536 to 319.250)	0.055			161.989 (28.423 to 295.556)	0.021
DNA damage signal	*H2AFX*			57.652 (−126.096 to 241.400)	0.512			−2.110 (−161.392 to 157.171)	0.978
	*RAD51*			−98.099 (−259.197 to 62.999)	0.213			−34.669 (−174.317 to 104.979)	0.603

Abbreviations: MPNST, malignant peripheral nerve sheath tumor; CI, confidence interval.

## Data Availability

The original contributions presented in this study are included in the article/Supplementary Materials. Further inquiries can be directed to the corresponding author.

## Supplementary Materials

The following supporting information can be downloaded at: https://www.mdpi.com/article/10.3390/biomedicines13020281/s1, Supplementary Material S1: Abbreviations of the genes used in this study. Supplementary Material S2: TPM values of the genes used in this study. Supplementary Material S3: *NELL2* activates *PAX7* during the malignant transformation of MPNSTs. Supplementary Material S4: *PAX7* activates *RAD52* during the malignant transformation of MPNSTs.
